# Large waterborne *Campylobacter* outbreak: use of multiple approaches to investigate contamination of the drinking water supply system, Norway, June 2019

**DOI:** 10.2807/1560-7917.ES.2020.25.35.2000011

**Published:** 2020-09-03

**Authors:** Susanne Hyllestad, Arild Iversen, Emily MacDonald, Ettore Amato, Bengt Åge Sørby Borge, Anton Bøe, Aslaug Sandvin, Lin T Brandal, Trude Marie Lyngstad, Umaer Naseer, Karin Nygård, Lamprini Veneti, Line Vold

**Affiliations:** 1 Department of Zoonoses, Food- and Waterborne Diseases, Norwegian Institute of Public Health, Oslo, Norway; 2 University of Oslo, Faculty of Medicine, Institute of Health and Society, Oslo, Norway; 3 Municipality of Askøy, Norway; 4 European Programme for Public Health Microbiology Training (EUPHEM), European Centre for Disease Control and Prevention (ECDC), Stockholm, Sweden; 5 Norwegian Food Safety Authority, Bergen, Norway

**Keywords:** disease outbreaks, Campylobacter, water supply, climate, drinking, Norway, extreme weather, gastroenteritis

## Abstract

On 6 June 2019, the Norwegian Institute of Public Health was notified of more than 50 cases of gastroenteritis in Askøy. A reservoir in a water supply system was suspected as the source of the outbreak because of the acute onset and geographical distribution of cases. We investigated the outbreak to confirm the source, extent of the outbreak and effect of control measures. A case was defined as a person in a household served by Water Supply System A (WSS-A) who had gastroenteritis for more than 24 h between 1 and 19 June 2019. We conducted pilot interviews, a telephone survey and an SMS-based cohort study of residents served by WSS-A. System information of WSS-A was collected. Whole genome sequencing on human and environmental isolates was performed. Among 6,108 individuals, 1,573 fulfilled the case definition. Residents served by the reservoir had a 4.6× higher risk of illness than others. *Campylobacter jejuni* isolated from cases (n = 24) and water samples (n = 4) had identical core genome MLST profiles. Contamination through cracks in the reservoir most probably occurred during heavy rainfall. Water supply systems are susceptible to contamination, particularly to certain weather conditions. This highlights the importance of water safety planning and risk-based surveillance to mitigate risks.

## Background

Campylobacteriosis is a common cause of bacterial diarrhoeal illness worldwide [1] and *Campylobacter jejuni* is the most common species in human infections [2]. Patients typically experience self-limiting diarrhoeal illness lasting 5 to 7 days [2]. Immunocompromised and elderly patients are at highest risk for prolonged illness and death [2]. Faecal-oral transmission to humans can occur through consumption of contaminated food and water, contact with animals and person-to-person contact [3]. In Norway, outbreaks of campylobacteriosis have been associated with consumption of untreated or contaminated drinking water, unpasteurised milk, mutton, contact with farm animals and with butchering, preparation and consumption of poultry [4-6].

Despite advances in water management and sanitation in high-income countries, waterborne outbreaks still occur and may acutely infect many people simultaneously [7]. Several waterborne outbreaks have been caused by contamination of the raw water source and inadequate hygienic barriers in the treatment process [8,9]. Updated regulations have improved safety at the treatment stage with a multiple-barrier approach in many water supply systems [10,11]. However, the distribution network is increasingly being identified as at risk for contamination through pipe breaks, cross connections and wastewater intrusion between the water treatment plant and the households [5,10,12,13]. Campylobacteriosis is the most commonly reported gastrointestinal disease in humans in Europe [14]. In Norway, waterborne outbreaks are detected every year [15,16], including two large waterborne outbreaks with more than 1,000 cases in the past 20 years. In 2004, an outbreak of giardiasis caused 1,300 confirmed cases and affected an estimated 6,000 residents in Bergen [17] and in 2007, an outbreak of campylobacteriosis associated with contaminated drinking water in Røros caused 1,500 cases [5].

## Outbreak detection

On the evening of 6 June 2019, the Medical Officer in Askøy reported an outbreak of gastroenteritis to the NIPH. In a 24 h period, 10 people had been hospitalised with fever, abdominal pain and diarrhoea, and ca 30 individuals had sought medical attention from out-of-hours primary healthcare services (OPHS). At least one person had tested positive for *Campylobacter*. Staff of the OPHS noted that many patients presenting with gastroenteritis had home addresses near each other, which led to a suspicion that drinking water could be the source of the outbreak. A joint investigation was carried out by the municipal services, the Norwegian Food Safety Authority and the Norwegian Institute of Public Health (NIPH), with the aim to confirm the source, extent of the outbreak and effect of control measures.

## Methods

### Outbreak context

The 29,500 inhabitants of the island municipality Askøy receive water from three different water supply systems, of which the largest, Water Supply System A (WSS-A) from the 1950s, serves ca 12,000 people in the south of the island. WSS-A has nine reservoirs, including three built as unlined mountain caverns. Its Reservoir X was early on suspected to be the source of the outbreak because of the geographical distribution of cases that clustered in two areas.

### Epidemiological investigations

#### Outbreak monitoring

In order to determine the extent of the outbreak, we collected data on in-person and telephone consultations with the International Classification of Primary Care (ICPC-2) codes for diarrhoea (D11), gastrointestinal infection (D70) and gastroenteritis (D73) that occurred at the OPHS and general practitioners’ (GP) offices in Askøy between 3 June and 15 June. We mapped all consultations by household address and water supply zone.

Several of the initial cases with confirmed *Campylobacter* infections were interviewed using a standardised 19-page trawling questionnaire in order to exclude possible exposures other than drinking water. The questionnaire included detailed questions about food consumption and purchases, animal contact and environmental exposures in the week before the onset of symptoms, as well as clinical and demographical information.

#### Survey of childcare centres

In order to rapidly ascertain the start and geographical areas of the outbreak, we contacted all childcare centres in the municipality on 11 and 12 June to document absence for illness. As children normally attend childcare centres close to their homes, it was likely that the household and childcare centre water supply zones would be the same. For the childcare centre survey, a case was defined as any person absent from the childcare centre (child or employee) because of the symptoms diarrhoea or vomiting between 28 May and 7 June. We then mapped the childcare centres by water supply zones served by the different reservoirs and compared the attack rates in childcare centres served by Reservoir X against those served by other reservoirs.

#### Cohort study of households

**Figure 1:**
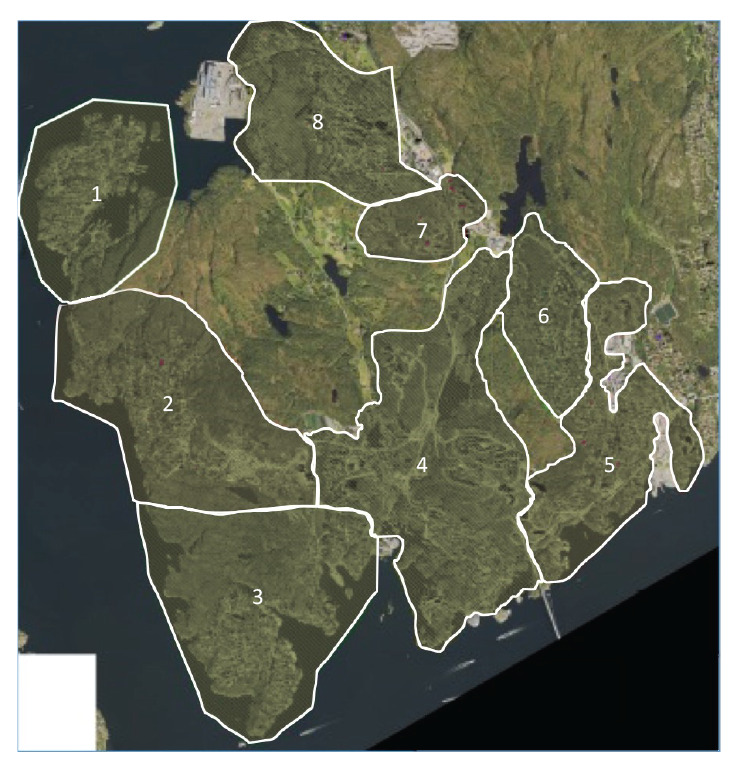
Water supply zones of water supply system WSS-A defined by different reservoirs, Askøy, Norway, 2019

We included all residents who received water from WSS-A in a retrospective cohort study, and identified eight different water supply zones (Figure 1).

Zones 6, 7 and 8 were served by Reservoir X.

People in households that partially or exclusively received water from Reservoir X were defined as exposed. We defined a case as a person with gastroenteritis (defined as having symptoms of (i) diarrhoea only or (ii) vomiting and at least one of the following: abdominal distention, fever, stomach pain or nausea, with duration of illness of more than 24 h), with symptom onset between 1 and 19 June 2019.

We sent all households served by WSS-A an SMS with a link to our questionnaire on 13 June 2019 and posted the link on the municipality’s website. We requested that one person should respond on behalf of all household members. We asked about household illness, clinical presentation, tap water consumption and whether the household had received and followed the boil water advisory (BWA). The survey was closed on 20 June.

We used R version 3.6.0 (2019–04–26) for statistical analyses.

### Microbiological investigations

The primary diagnostic laboratory sent *Campylobacter* isolates from patients to the National Reference Laboratory for Enteropathogenic Bacteria (NRL) for confirmation and genotyping.

We extracted DNA on MagNAPure 96 (Roche Molecular Systems Inc., Pleasanton, United States (US)) and used KAPA HyperPlus (Kapa Biosystems, Wilmington, US) for library preparation and Agencourt AMPure XP (Beckmann Coulter Life Sciences, Indianapolis, US) for removal of adaptor dimers. Whole genome sequencing was performed as paired-end (250 bp × 2) sequencing on the MiSeq platform (Illumina Inc., San Diego, US) aiming for coverage of > 50×. Quality control of the raw reads was done through FastQC.

We used SeqSphere+ software, version 5.1 (Ridom GmbH, Münster, Germany) for analysis. Briefly, multilocus sequence typing (MLST) of the *Campylobacter* isolates was performed using the seven-gene scheme developed by Keith Jolly at PubMLST [18]. Core genome MLST was performed using SeqSphere+ integrated scheme for *Campylobacter* of 637 core and 958 accessory genome targets modified from Cody et al. [19,20].

According to the routine sampling and analysis plan for WSS-A, water samples are collected and tested for faecal indicator bacteria: weekly for *Escherichia coli* and coliform bacteria and for heterotrophic plate count, and monthly for intestinal enterococci and *Clostridium perfringens*, according to standard methods described in the national drinking water legislation [21].

After the outbreak was detected, we started extra sampling of the water in WSS-A and analysed it for faecal indicator bacteria at an accredited laboratory using standard methods. On 6 June, we also took water samples from Reservoir X and several other points along the distribution system and immediately analysed it for *Campylobacter* using semiquantitative and quantitative determination in foods and drinking water (NMKL 119, 3.Ed., 2007) with pre-incubation on enrichment broth of filtered sample followed by plating on a selective medium [22]. The presence of *Campylobacter* was confirmed by phase contrast microscopy.

### Environmental investigations

We reviewed operational system information and historical data from WSS-A, including results from drinking water routine monitoring schemes before the outbreak. We assessed critical points and possible sources of contamination (including system failures and unusual events) through interviews of WSS-A staff and visual inspections of selected areas of WSS-A, including Reservoir X. Local rainfall was assessed using data from the national meteorological website (www.yr.no).

### Ethical statement

Approval by a regional committee for medical research was not needed as the NIPH is authorised to access and use personal identifiable information for communicable disease outbreak investigations in the public interest. All persons invited to the pilot interviews and childcare centres study provided oral consent to be a part of the study. They were informed that they could withdraw at any time during the study and that their data would be deleted. The SMS questionnaires were distributed by Askøy municipality and personal identifiers were not a part of the dataset.

## Results

### Epidemiological investigation

#### Outbreak monitoring and timeline of action points

A BWA was issued when the outbreak was detected on 6 June. Reservoir X was taken out of service on 7 June and inspected on 9 June by representatives from Askøy municipality and NIPH. The pilot interviews and childcare centre survey were conducted on 11 June, and the SMS-based cohort study started on 13 June.

**Figure 2:**
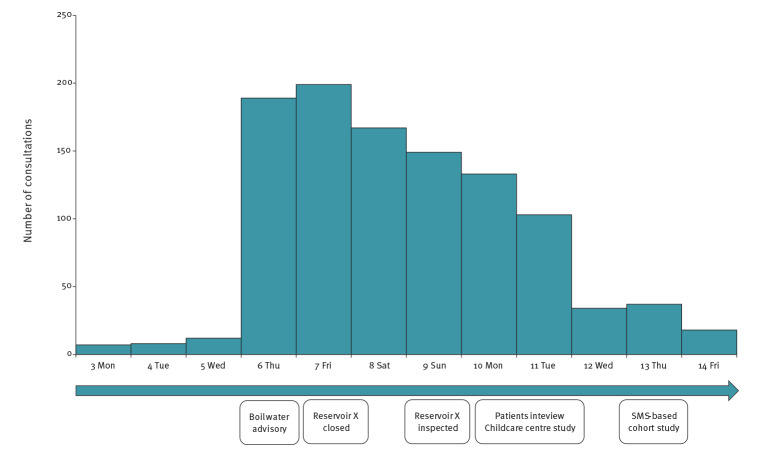
Number of gastroenteritis consultations (including telephone consultations) at OPHS and GP offices, Askøy, Norway, 3–14 June 2019 (n = 1,056) and timeline of action points

GP: general practitioner; OPHS: out-of-hours primary healthcare services.

Data collected at the OPHS and GP offices revealed a sharp increase in the number of consultations for gastroenteritis (from 12 to 182 consultations) on Thursday 6 June (Figure 2). The consultations were evenly distributed among all age groups, although in-person consultations were primarily for children.

**Figure 3:**
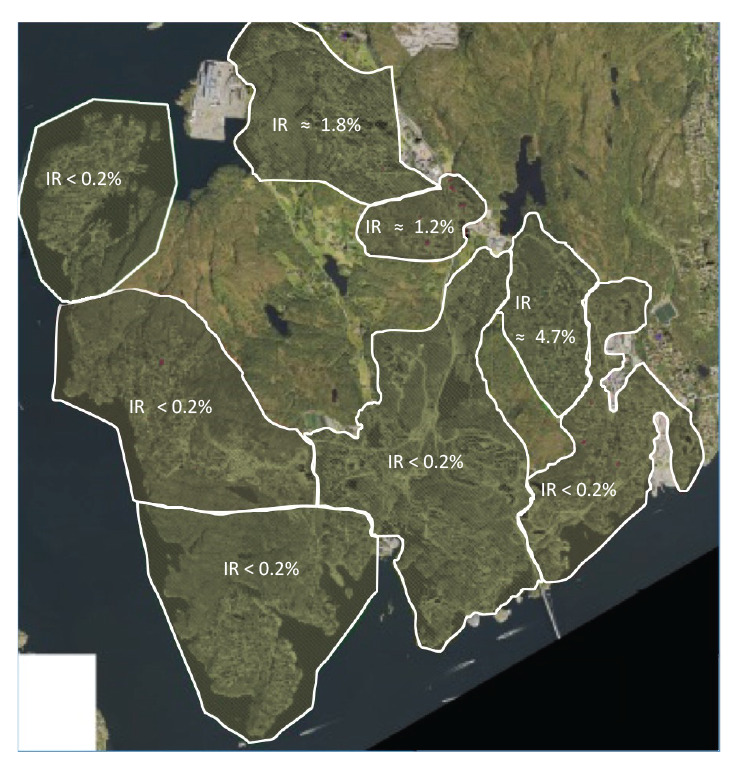
Estimated incidence rates for gastroenteritis consultations linked to reservoir supply zones, Askøy, Norway, 6 June 2019

The gastroenteritis patients’ residences were geographically concentrated in two areas of the municipality, which coincide with three water supply zones served by Reservoir X. These zones had higher incidence rates (IR) for consultations than other supply zones in WSS-A at the time of the outbreak detection (Figure 3).

IR: incidence rate.

The three zones with IR > 1 are the ones served by Reservoir X.

#### Pilot interviews with confirmed cases

We interviewed five of the first cases with confirmed *Campylobacter* infection. They reported diarrhoea, stomach pain and fever with onset on 4 June (n = 1) or 5 June (n = 4). They lived in areas that received drinking water from WSS-A and had consumed tap water at home or used tap water for brushing teeth in the week before symptom onset. Other reported exposures, such as attendance at common dinners or events, consumption of food items, contact with animals or recreational water exposure, were not common to all five cases.

#### Survey of childcare centres

All 27 childcare centres in the municipality participated in the study; eight (with 769 children and employees) were in areas supplied by Reservoir X and 19 (with 1,761 children and employees) were in areas supplied by other reservoirs. The overall attack rate was 20% for the childcare centres in affected areas and 2% for the childcare centres in unaffected areas. Absences started to increase at the childcare centres in affected areas on Monday 3 June (n = 26) and peaked on Friday 7 June with 81 absences (11%).

#### Cohort study of households

The SMS with the questionnaire was sent to 4,409 persons with mobile phone numbers registered to residences in the supply area of WSS-A. Data from 6,192 individuals were reported through the online questionnaire, of whom 1,913 reported illness. We excluded data for 79 household members who reported onset of illness before 1 June and for five who reported onset of illness after 19 June. After this exclusion, data were available from 2,526 persons who responded on behalf of 6,108 household members, which yields a coverage of 51% (6,108/11,995) of the residents supplied by WSS-A. Mean age of the included household members was 34 years (range: 0 to 93 years) and 50% were female.

**Figure 4:**
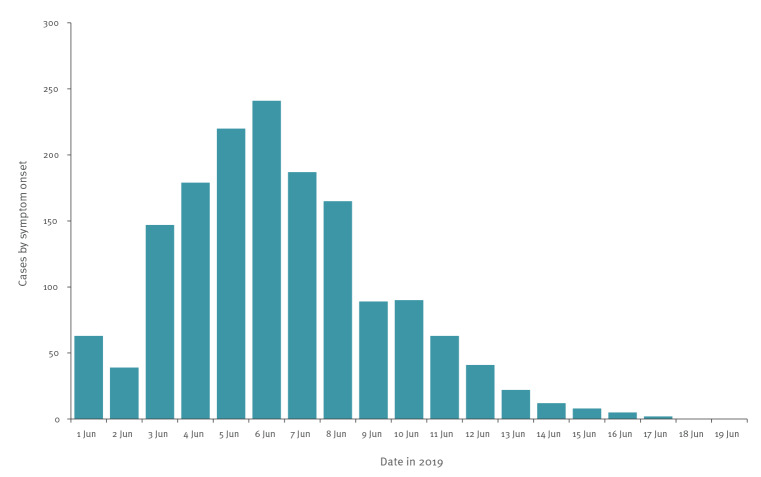
Distribution of cases by date of symptom onset, Askøy, Norway, 1–19 June 2019 (n = 1,573)

A total of 1,829 persons reported at least one of the following symptoms: diarrhoea (n = 1,626; 89%), abdominal pain (n = 1,347; 74%), headache (n = 959; 52%), nausea (n = 935; 51%), fever (n = 868, 48%), abdominal distention (n = 639; 35%), vomiting (n = 286; 16%) and bloody stool (n = 113; 6%). In total, 1,573 respondents met the case definition, leading to an attack rate of 26%. The number of cases by date of symptom onset peaked on 6 June and decreased gradually thereafter (Figure 4). The mean duration of symptoms was 4.6 days (range: 2–16 days).

Most respondents sent their response on 13 June, numbers after this date may be uncertain.

Residents who were supplied from Reservoir X had a 4.6× higher risk of illness than residents served by other reservoirs in WSS-A (Table 1).

**Table 1:** Estimated attack rates and risk ratio for areas supplied by Reservoir X and other areas, gastrointestinal illness, Askøy, Norway, 2019 (n = 6,108)

Reservoir	Households	Individuals	Cases	Attack rate	Risk ratio (95% confidence interval)
Other reservoirs in WSS-A (zones 1–5)	1,653	4,098	481	12%	Reference
Reservoir X (zones 6–8)	873	2,010	1,092	54%	4.6 (4.2–5.0)

Among residents supplied by Reservoir X, the risk of illness increased with the amount of daily consumption of tap water (p value < 0.001) (Table 2).

**Table 2:** Risk of gastrointestinal illness by consumption of tap water, Askøy, Norway, 2019 (n = 6,108)

Daily tap water consumption	Individuals	Cases	Attack rate	Risk ratio (95% confidence interval)
0 glasses	381	27	7%	Reference
1–3 glasses	2,562	586	23%	3.2 (2.2–4.7)
4–6 glasses	2,255	654	29%	4.1 (2.8–5.9)
≥ 7 glasses	910	306	34%	4.7 (3.3–6.9)

Regarding the notification of BWA via SMS, 88% of households (2,223 of 2,526) reported that they had received an SMS with the BWA, while 179 (7%) did not receive the BWA and 124 (5%) were unsure.

A total of 2,384 households reported having complied with the BWA (compliance rate: 95%: 2,384/2,526). Reasons for non-compliance were reported by 142 of the households; the main reasons were purchasing bottled water (n = 76), considering that the risk of becoming ill was low (n = 9) and drinking little or no tap water (n = 4). For the remaining 53 households, reasons for non-compliance to the BWA were not reported. 

### Microbiological investigation

#### Analysis of *Campylobacter*

The Norwegian Surveillance System for Communicable Diseases (MSIS) registered 181 laboratory-confirmed cases of campylobacteriosis linked to this outbreak. The NIPH received 24 isolates of *C. jejuni* from persons served by Reservoir X. All isolates were sequence-typed as ST1701, which is part of the clonal complex ST-45, and all had identical cgMLST profiles, cluster type 97 (0 allelic differences). Two representative sequences have been submitted to the European Nucleotide Archive (ENA) (ERS4574581, ERS4574582). When comparing publicly available ST1701 *C. jejuni* sequences to the outbreak strain, we did not observe any close genetic relationships, although poultry or birds were identified as a possible source.

#### Analysis of water

In the 3 years before 3 June 2019, the routine monitoring programme for WSS-A did not detect any faecal indicator bacteria in WSS-A or in Reservoir X, except for occasional findings of coliform bacteria. The samples collected during routine monitoring for WSS-A on 3 June were also negative for faecal indicator bacteria, including samples located near Reservoir X.

Of eight water samples taken on 6 June, five were collected from Reservoir X and areas supplied by Reservoir X and were faecally contaminated, while the remaining three samples from other areas were not. Seven water samples collected from the distribution system at the same locations on 9 June, after Reservoir X was taken out of service on 7 June, were negative for faecal indicator bacteria. One sample taken inside Reservoir X on 9 June was positive for faecal indicator bacteria, as were all samples collected in Reservoir X during the week of the outbreak (until 13 June).

Four of seven water samples were positive for *Campylobacter* (7 June). The positive samples had been taken from Reservoir X, two households and one school supplied by Reservoir X.

### Environmental investigation

#### Description of the water supply network

Under normal conditions, Reservoir X supplies a defined zone (zone 6) of 1,350 residents. However, before the outbreak, a valve had been opened from Reservoir X to ensure replacement of water in response to customer complaints about the water quality in the area. This led to a connection between zone 6 and zones 7 and 8 (Figure 1), serving in total 3,558 residents with drinking water from both Reservoir X and other reservoirs. Zone 6, 7 and 8 were all considered zones affected by water supplied by Reservoir X and consultations indicated a higher IR in these zones initially in the outbreak detection (Figure 3). The valve was closed on 6 June.

#### Visual inspection of Reservoir X

Reservoir X is a basin constructed as an unlined rock cavern with an entrance sealed by a locked door. It holds ca 400 m^3^ of water and is located above a residential area in mountainous terrain. The inflow and outflow of Reservoir X is through the same pipe. Because of the design of the reservoir, the chosen sampling location for routine monitoring samples did not capture the water flowing out of the reservoir. Potential contamination introduced to the reservoir would therefore not necessarily not be detected.

During the visual inspection of Reservoir X, we observed natural cracks located in the back of the reservoir. There were leaks in the concrete construction and we observed water running from inside the roof. A large antenna is also located above the reservoir, with power lines running over the closed cavern, were birds could potentially gather and increase the risk of bird faeces contaminating the area below. Although varied wildlife is reported in the area, we did not observe this during our inspection of the area.

**Figure 5:**
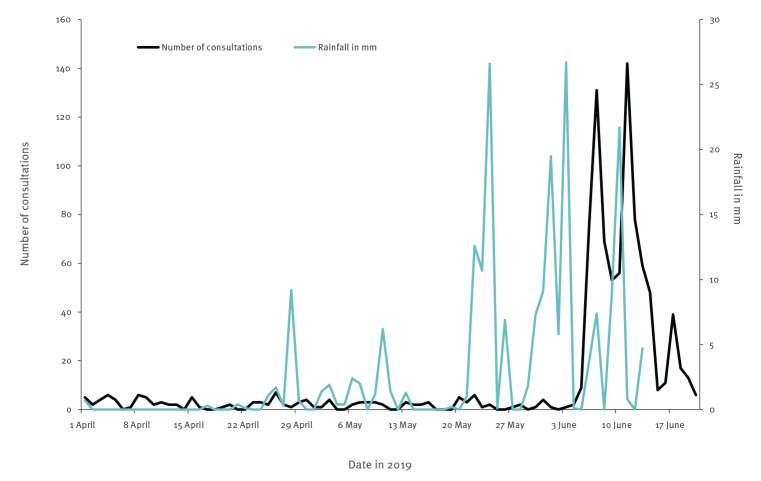
Data on rainfall from a nearby weather station and onset of consultations for gastroenteritis registered in the Norwegian Syndromic Surveillance System (NorSySS), Askøy, 1 April–20 June 2019 (n = 948)

No unusual malfunctions in the distribution system were reported before the outbreak. In weeks 21 and 22, three episodes of extreme rainfall (24 May, 1 June and 3 June) occurred in the area after a long dry period from 1 April to 20 May. Weather data obtained from a nearby weather station (Skredderdalen) indicated heavy rainfall which coincided with registered consultations of gastroenteritis in the Norwegian Syndromic Surveillance System (NorSySS) (Figure 5).

## Outbreak control measures

In addition to issuing a BWA and closure of Reservoir X (Figure 1), emergency water supply distribution started on 8 June from water tanks located in public areas such as school and parking lots, and infection control measures in public services were strengthened, as a measure to address the concern in the population.

## Discussion

The results of epidemiological, microbiological and environmental investigations support that contaminated water from Reservoir X caused an outbreak of more than 1,500 cases of campylobacteriosis in Askøy in June 2019. The triangulation of epidemiological, genomic, geographical and water systems data was essential for confirming the role of Reservoir X in the outbreak and determining the extent of exposure within the community. The rationale for the decisions early in the phase of the outbreak was based on local knowledge and mapping of cases rather than epidemiological studies. This observation gave a hint towards the drinking water supply system as the source to the outbreak. The use of multiple approaches and use of mixed methods also allowed us to identify contributing factors, such as inclement weather conditions, and ensure that the implemented control measures had successfully stopped the outbreak. While we were unable to conclusively determine how the reservoir became contaminated, the diversity of data sources used to investigate this outbreak support the hypothesis that environmental contamination through cracks in Reservoir X most likely occurred during heavy rainfall following a long dry period.

*Campylobacter* has frequently been identified as the cause of waterborne outbreaks [10,23], often associated with heavy rainfall [24,25] and intrusion of contaminated surface water either into source water [26-30] or into the distribution network [31,32]. In 2006, a risk analysis of WSS-A pointed out the vulnerability of unlined reservoirs established in mountains. Reservoir X was therefore scheduled to be taken out of service, as a part of long-term precautionary actions, and replaced by a newly built reservoir in the area in February 2019, several months before the outbreak. However, work to connect the new reservoir was delayed and only a limited number of households had been connected to the new reservoir at the time of the outbreak. Given the identified vulnerabilities, the occurrence of an outbreak under these conditions was foreseeable [33] and serves as a reminder that the drinking water is susceptible for contamination at any time, even during transition phases [5,17]. Continuous risk assessment, followed by implementation of long-term precautionary actions, is essential to protect the drinking water from contamination, while simultaneously ensuring day-to-day operation [34].

There was no indication of contamination with faecal indicator bacteria before the outbreak from routine sampling conducted on 3 June. This is a common finding in many waterborne outbreaks in which routine monitoring was neither the source of early detection nor able to prevent the occurrence of an outbreak [7]. Although traditional routine monitoring conducted by the waterworks serves an important function in terms of assessing the performance of the water supply system, it does not reliably allow for monitoring imminent health risks in distribution systems, surveillance of diseases or prediction of outbreaks [35,36]. To allow early detection of deviant water quality in the distribution system, technological advances are being made in terms of real-time monitoring of water distribution supply systems. However, it may still be difficult to link the results to public health monitoring systems and operational response [37]. This highlights the need to focus on water safety planning to protect the water supply from contamination and to conduct risk-based surveillance, rather than detecting the contamination in retrospect [38]. This is particularly relevant when external risk factors to the water supply systems are changing, such as unexpected rainfall patterns, and the existing infrastructure may not be designed to adapt or is vulnerable because of lack of maintenance and upgrading.

Timely detection of waterborne outbreaks is crucial in preventing widespread exposure of the population and limiting the negative health consequences. Although the exposure to contaminated drinking water may be almost instantaneous for the supplied residents, the detection may be delayed, particularly in large areas served by the same water supply [17,39], while the time between exposure and detection may be shorter in more limited contexts [5]. To overcome delays in detection that may occur when relying on surveillance of laboratory-confirmed cases, syndromic surveillance of real-time (or near to real-time) clinical signs and symptoms as well as proxy measures [40,41] may be useful, especially if health data can be linked to water supply system information [42,43]. In Askøy, the outbreak alert was generated by astute healthcare workers on the evening of 5 June and on 6 June, information on the geographical distribution of the consulting patients’ addresses were manually plotted on maps, demonstrating a link to Reservoir X. Although there are theoretical benefits of a surveillance system based on epidemiological indicators, either on an ongoing basis or triggered by spatio-temporal exceedances it is unlikely that this would have led to a more timely detection in Askøy. However, having systems in place that allow for rapid combined analysis of health and water systems data may have simplified the investigation of the outbreak and monitoring of the efficacy of the control measures.

This investigation has several limitations. Initially, it was difficult to determine the scale of the rapidly developing outbreak. Although campylobacteriosis is a notifiable disease in Norway, only a small proportion of sick residents were tested and laboratory-confirmed cases were not reported rapidly enough to follow the progression of the outbreak. For this reason, consultations for gastroenteritis were tracked manually through the GP and OPHS offices on a daily basis for outbreak monitoring until monitoring with NorSySS was possible. The SMS-based cohort study corroborated the onset and duration of the outbreak, but since only one person in the household was asked to respond on behalf all members of the family, this may have led to an underestimation of cases. In addition, the specific case definition excluded more than 500 people who reported illness possibly linked to the outbreak. On the other hand, the widespread media attention may have affected the results of the SMS-based cohort study by overestimating illness among the respondents. However, we chose to use a specific case definition to avoid including people that were ill for other reasons.

## Conclusions

A large waterborne outbreak leading to more than 1,500 cases of campylobacteriosis occurred in Askøy in Norway in June 2019. Through multiple data sources, we were able to determine that contamination of drinking water occurred through cracks in a mountain reservoir, probably because of heavy rainfall after an extended dry period. This is a reminder that water supply systems, in particular ageing infrastructure, are generally vulnerable and susceptible to contamination that may result in an outbreak, especially as external risks such as climate factors are changing. This investigation highlights the importance of conducting water safety planning, updating the infrastructure and performing risk-based surveillance to mitigate risks.
