# Author’s correction for Euro Surveill. 2020;25(35)

**DOI:** 10.2807/1560-7917.ES.2020.25.43.201029c2

**Published:** 2020-10-29

**Authors:** Eurosurveillance editorial team

**Affiliations:** 1 European Centre for Disease Prevention and Control (ECDC), Stockholm, Sweden

In the article “Large waterborne Campylobacter outbreak: use of multiple approaches to investigate contamination of the drinking water supply system, Norway, June 2019" by Hyllestad et al, published on 3 September 2020, an addition was made on request of the authors: The sentence “*Campylobacter jejuni* isolates from cases (n = 24) and water samples (n = 4) were identical by WGS” was added to the chapter on the Microbiological investigation. These changes were published on 28 October 2020.

